# A clinical observation of carbon dioxide laser-assisted deep sclerectomy for Posner-Schlossman Syndrome

**DOI:** 10.3389/fmed.2026.1623445

**Published:** 2026-02-04

**Authors:** Tao Luo, Jun Jiang, Sanni Yang, Heng Liu, Yang Shen, Yan Wu, Yuancheng Zhao, Pan Long, Fei Han

**Affiliations:** 1 Department of Ophthalmology, The General Hospital of Western Theater Command, Chengdu, China; 2 School of Materials Science and Engineering, Southwest Jiaotong University, Chengdu, China

**Keywords:** carbon dioxide laser-assisted deep sclerectomy surgery, intra-ocular pressure, Posner-Schlossman Syndrome, scleral pool, secondary glaucoma

## Abstract

**Objective:**

This study aimed to preliminarily evaluate the clinical efficacy and safety of carbon dioxide laser-assisted deep sclerectomy (CLASS) in patients with medically refractory Posner-Schlossman Syndrome (PSS).

**Methods:**

Six patients (six eyes) with poorly controlled PSS were included. All patients underwent CLASS under local anesthesia and were followed up for 18 months. Primary outcome measures included intraocular pressure (IOP), best-corrected visual acuity (BCVA), retinal nerve fiber layer (RNFL) and ganglion cell complex (GCC) thickness, visual field parameters, and surgical complications.

**Results:**

The mean preoperative IOP was 28.30 ± 5.16 mmHg. Postoperative IOP decreased significantly, with a mean IOP of 14.56 ± 1.82 mmHg at the final 18-month follow-up. Visual acuity recovered to or exceeded preoperative levels in all cases. No statistically significant differences were observed in RNFL or GCC thickness at any postoperative time point (6, 12, and 18 months) compared to preoperative values. Visual field indices (MD, PSD) remained stable throughout follow-up. Postoperative complications included transient hypotony (three eyes), peripheral anterior synechiae (two eyes, successfully treated with laser), transient shallow anterior chamber (one eye), and mild anterior chamber reaction (one eye). All complications were managed conservatively without sequelae, and no serious adverse events occurred.

**Conclusion:**

CLASS surgery provides effective and sustained IOP reduction in patients with refractory PSS. It demonstrates favorable structural and functional preservation of the optic nerve over the mid-term follow-up, with a manageable profile of minor complications. These preliminary findings suggest CLASS is a promising minimally invasive surgical option for this condition, though its long-term efficacy warrants confirmation in larger prospective studies.

## 1 Introduction

Posner-Schlossman Syndrome (PSS) is a distinct clinical entity, typically unilateral, that manifests as recurrent non-granulomatous anterior uveitis accompanied by marked intraocular pressure elevation, representing a specific subtype of glaucomatous anterior uveitis (1). PSS is characterized by recurrent episodes of markedly elevated intraocular pressure (IOP) during acute uveitis, with IOP typically normalizing between attacks. It primarily affects young and middle-aged adults (20–50 years old) and accounts for 1.8%−6.7% of all uveitis cases annually. While its etiology remains unclear, potential contributing factors include inflammation, infection, and dysregulated immune responses. Patients with PSS typically exhibit open anterior chamber angles. Some clinical studies suggest that the acute IOP elevation may result from both increased aqueous humor production and decreased outflow facility. The condition generally responds well to treatment with glucocorticoids and IOP-lowering medications (2). However, a subset of patients experiences recurrent disease that is not adequately controlled in time. This can result in the development of glaucomatous optic nerve and visual field damage, at which point surgery is required.

Conventional surgical interventions for glaucoma primarily encompass trabeculectomy (often combined with peripheral iridectomy), ciliary body ablation, and glaucoma drainage device implantation. Given that PSS presents as a secondary open-angle glaucoma, the standard approach in practice has been trabeculectomy with peripheral iridectomy, which can provide satisfactory IOP control for some patients (3). Nevertheless, most patients with PSS are young or middle-aged, a demographic prone to more pronounced postoperative adhesions. Scarring of the scleral flap and conjunctival filtration bleb is frequently observed, which can obstruct aqueous outflow and lead to recurrent IOP elevation, ultimately compromising the long-term efficacy of the procedure. Furthermore, as PSS is associated with underlying inflammatory activity, intraocular maneuvers such as iridectomy may exacerbate inflammation. This inflammatory response can promote trabecular meshwork blockage and angle closure, further aggravating IOP dysregulation. Standard trabeculectomy can be associated with complications including anterior chamber hemorrhage, shallow anterior chamber, choroidal detachment, and fibrosis/scarring of the filtration bleb. By contrast, the CLASS technique employs laser ablation to selectively thin deep scleral tissue. This approach can significantly reduce the risk of scarring and markedly lower the incidence of postoperative shallow anterior chamber, while maintaining anterior chamber integrity (4).

Among the newer surgical options for glaucoma, carbon dioxide (CO_2_) laser-assisted deep sclerectomy (CLASS) has emerged as a promising technique and is increasingly adopted in clinical practice, owing to its minimally invasive nature, relative technical ease, and favorable complication profile (5). CLASS surgery evolved from conventional non-penetrating filtering surgery (NPTS), with the key modification being the integration of CO_2_ laser technology. This allows for precise ablation of the deep sclera and the outer wall of Schlemm's canal while maintaining the integrity of the eye wall (6). This technique leverages the property of CO_2_ laser energy being highly absorbed by water. The laser is used to precisely ablate the deep sclera and the outer wall of Schlemm's canal, avoiding full-thickness penetration of the eyeball. This process reduces aqueous outflow resistance, establishes a new drainage pathway, and thereby effectively lowers intraocular pressure (7). Interestingly, the efficacy of IOP control is not determined by the size of the postoperative scleral pool. UBM evaluations reveal a poor correlation between pool dimensions and IOP, contrasting with a positive association between IOP and the thickness of the posterior elastic layer–trabecular meshwork complex. The concurrent detection of hypoechoic areas in the suprachoroidal space implies aqueous drainage through the attenuated deep sclera into this region. Thus, even with an ill-defined scleral pool, the choroidal pathway remains a critical contributor to IOP lowering (8, 9). CO_2_ laser assistance markedly lowers the risk and procedural complexity inherent in traditional NPTS. Although CLASS surgery does not achieve greater IOP reduction than trabeculectomy, it holds a superior profile regarding lower rates of early postoperative complications, better corneal endothelial safety, and easier reparability. This report presents a case series in which CLASS was employed to treat six patients with PSS.

## 2 Information and methodology

### 2.1 General information

Six patients (six eyes) diagnosed with Posner-Schlossman Syndrome (PSS) between January 2019 and June 2020 were included in this study. The cohort comprised four males (four eyes) and two females (two eyes), with two right eyes and four left eyes involved. The mean age was 26.4 years (range: 16–45 years). All patients presented with mild conjunctival congestion. Anterior chamber examination revealed normal depth and clarity with a Tyndall effect graded as negative [Tyn (–)]. Corneas were transparent, each exhibiting one – two well-demarcated keratic precipitates (KPs) on the endothelial surface. Pupils were round with intact light reflexes, and no iris adhesions were observed. Ocular ultrasound showed no significant abnormalities. After excluding other causes of secondary glaucoma, all patients had received topical corticosteroids and a combination of at least two different classes of IOP-lowering medications (including timolol, brinzolamide, brimonidine tartrate, and/or travoprost) yet demonstrated persistently poor IOP control. The mean preoperative IOP under medication ranged from 26 to 35 mmHg. Preoperative best-corrected visual acuity (BCVA) was 0.1–0.3 in three eyes, 0.5 in one eye, and >0.5 in two eyes. Inflammation was considered clinically stable at the time of surgery, characterized by the absence of active KPs, no increase in KP number, a clear anterior chamber, Tyn (–), and no obvious fundus abnormalities (see Table 1 for a summary). Preoperative assessment confirmed controlled anterior segment inflammation [clear anterior chamber, Tyn (–)] and ruled out other secondary glaucoma etiologies in all patients.

**Table 1:** Baseline demographic and clinical characteristics of the six patients with Posner-Schlossman Syndrome (PSS) who underwent CLASS surgery.

**Patient no**.	**Sex**	**Age (years)**	**Eye**	**Duration of PSS (years)**	**Preoperative IOP on medications (mmHg)**	**Number of preoperative IOP-lowering medications**	**Preoperative BCVA**	**Number of KP**	**Iris adhesion**	**Follow-up (months)**
1	Male	28	OS	3	32	3	0.2	2	No	18
2	Female	45	OD	5	35	3	0.5	1	No	18
3	Male	22	OS	2	28	2	0.1	2	No	18
4	Male	16	OD	1	26	2	0.6	1	No	18
5	Female	24	OS	4	30	3	0.3	2	No	18
6	Male	23	OS	2	29	2	0.8	1	No	18

IOP, intraocular pressure; BCVA, best-corrected visual acuity; KP, keratic precipitates; Eye: OD (oculus dexter, right eye); OS (oculus sinister, left eye).

### 2.2 Surgical procedure

All surgeries were performed under local anesthesia by the same surgeon. One day prior to surgery, topical pilocarpine (2%) was administered six times to induce miosis. Intraoperatively, subconjunctival infiltration anesthesia was achieved using 2% lidocaine. A fornix-based conjunctival flap was created in the superior quadrant. A partial-thickness (approximately one-third to one-half scleral depth) rectangular scleral flap (5 × 5 mm) was dissected and extended 1 mm anteriorly into clear cornea. Mitomycin C (MMC; 0.4 mg/mL) was applied beneath the scleral flap for 3–5 min, followed by copious irrigation with balanced salt solution. A 4 × 2 mm deep scleral pool was created within the bed of the superficial flap using a CO_2_ laser (OcuLight^®^ TX; IOPtima Ltd., Tel Aviv, Israel) set at 19–21 W (Begin with the lowest energy level and gradually increase it based on the tissue's response.) in continuous-wave mode. Ablation was performed meticulously until the underlying uveal pigment was visible, aiming for maximal depth without perforation. Following this, a fresh MMC-soaked sponge (0.4 mg/ml) was reapplied to the bed for 30 s to 2 min. Using the same laser with a curved aiming beam (4 × 1.2 mm) focused on the gray line of the corneal limbus, the outer wall of Schlemm's canal was ablated with a 2-s pulse interval. Ablation was continued until aqueous percolation was observed. The scleral flap was then repositioned and secured with four interrupted 10-0 nylon sutures, followed by closure of the conjunctival incision with the same suture material. At the conclusion of surgery, tobramycin and dexamethasone ointment (Tobradex^®^) was applied. Postoperative regimen consisted of topical tobramycin-dexamethasone eye drops four times daily for 2 weeks, tapered over the subsequent month, and timolol maleate 0.5% eye drops twice daily for 3 months.

### 2.3 Examinations and follow-up

Patients were included if they met all the following criteria: a definitive diagnosis of PSS with recurrent anterior uveitis involving the iris and/or ciliary body; IOP persistently exceeding 21 mmHg despite treatment with at least two different classes of topical IOP-lowering medications; quiescent intraocular inflammation at the time of surgery, characterized by the absence of anterior chamber exudate, clear keratic precipitates, and an anterior chamber cell count of ≤ 5 cells within a standardized 1 mm × 1 mm slit-lamp beam field; and good general health. Exclusion criteria comprised the presence of neovascular glaucoma, active uncontrolled intraocular inflammation, other forms of secondary glaucoma, or a history of previous incisional glaucoma surgery in the study eye. Surgical success was defined as the maintenance of postoperative IOP between 10 and 21 mmHg at the final follow-up visit (18 months), with cases categorized as complete success (without adjunctive IOP-lowering medication) or qualified success (with the use of such medication).

All patients underwent comprehensive ophthalmic examinations preoperatively and at scheduled postoperative visits on day 1, day 7, and at 1, 3, 6, 12, and 18 months. Intraocular pressure was measured three times using a non-contact tonometer (Reichert, USA) and the average value was recorded. Best-corrected visual acuity (BCVA) was assessed with a standard logarithmic visual acuity chart and converted to LogMAR units for analysis. Anterior segment imaging via ultrasound biomicroscopy (UBM) was performed to evaluate the morphology of the scleral pool and filtration structures. Posterior segment assessment included spectral-domain optical coherence tomography (SD-OCT) to measure the thickness of the ganglion cell complex (GCC) and the peripapillary retinal nerve fiber layer (RNFL). Visual field testing was conducted using standard automated perimetry.

### 2.4 Statistical analysis

Given the longitudinal nature of the data, repeated-measures analysis of variance (ANOVA) was used as the primary method to analyze changes over time in IOP, GCC thickness, and RNFL thickness. The effect size for these comparisons was calculated using partial eta-squared (η^2^), with values interpreted as small (η^2^ ≥ 0.01), medium (η^2^ ≥ 0.06), and large (η^2^ ≥ 0.14). The results are presented as mean ± standard deviation, with 95% confidence intervals (CI) and corresponding *p-*values. However, it is acknowledged that the statistical power of parametric tests like ANOVA is limited in small-sample pilot studies such as this. Therefore, the non-parametric Friedman test was also employed as a complementary analysis to assess overall differences across time points, regardless of distributional assumptions. The significance level was set at *p* < 0.05 for all tests. Statistical analyses were performed using SPSS software (IBM Corp., Armonk, NY, USA).

## 3 Results

### 3.1 Visual acuity and visual field

Postoperative visual acuity showed a transient decline in three eyes at day 1, which recovered to baseline levels within 1 week. By 1 month, all six eyes had regained their preoperative visual acuity. Thereafter, visual acuity remained stable: one eye showed an improvement of one Snellen line at the three- and six-month visits, while the others maintained their preoperative levels. No significant change in visual acuity was observed between the six- and 12-month follow-ups, and all visual fields remained stable compared to preoperative baseline at both 6 and 12 months postoperatively.

### 3.2 Intraocular pressure (IOP)

**Figure 1:**
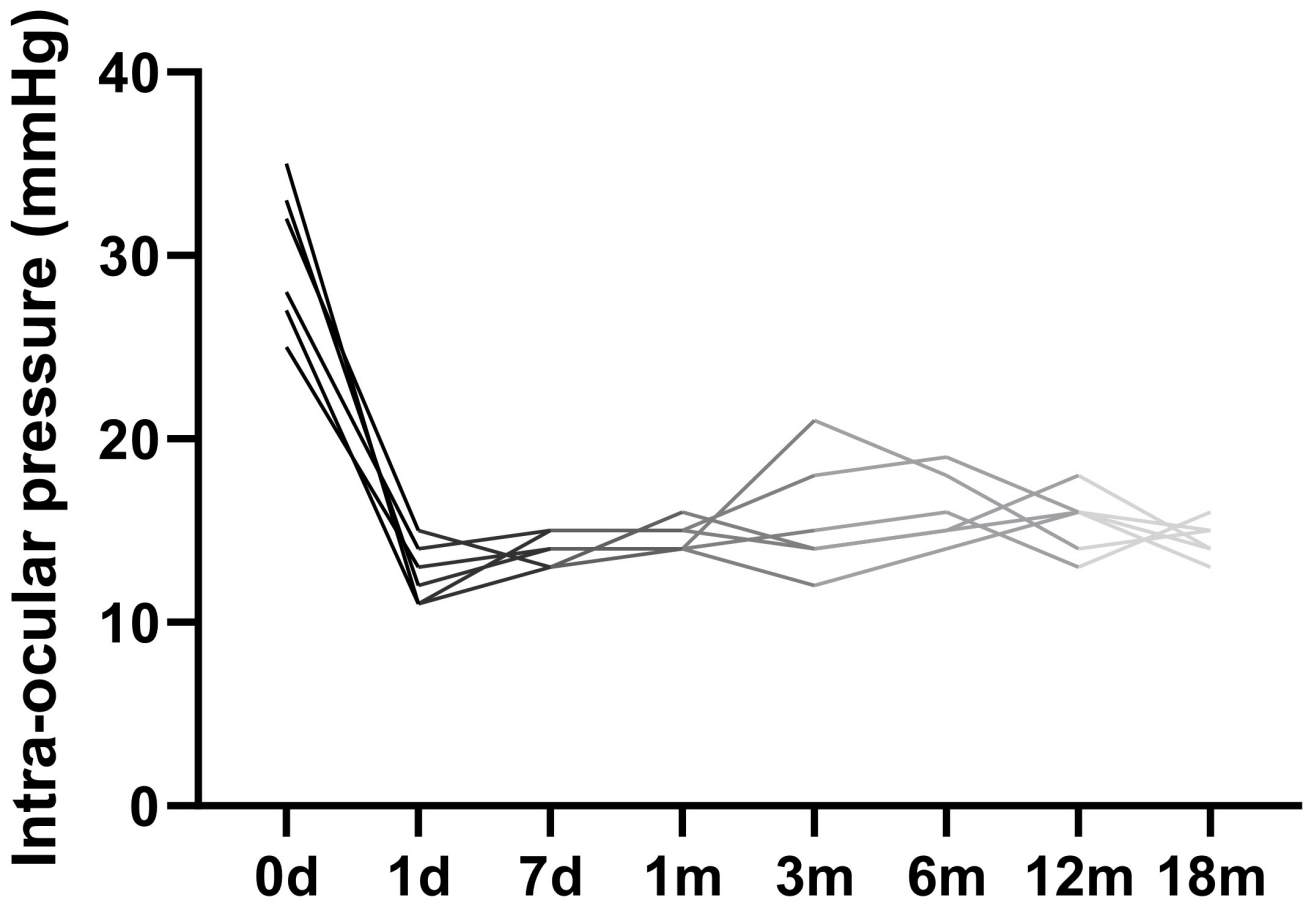
Intraocular pressure (IOP) measurements over the preoperative and 18-month postoperative period.

Intraocular pressure (IOP) showed a significant reduction following CLASS surgery (Figure 1). The mean preoperative IOP was 28.30 ± 5.16 mmHg. Postoperatively, mean IOP decreased to 12.32 ± 0.98 mmHg at day 1, 12.17 ± 0.75 mmHg at day 7, 14.00 ± 1.26 mmHg at 1 month, 15.50 ± 1.52 mmHg at 3 months, 14.67 ± 1.21 mmHg at 6 months, 14.33 ± 1.37 mmHg at 12 months, and 14.56 ± 1.82 mmHg at 18 months, indicating sustained control. A mild IOP rebound was observed in two eyes at the three- and six-month visits; however, their pressures remained below preoperative levels. Overall, IOP stabilized in all eyes by the 12-month follow-up.

### 3.3 Ganglion cell complex and retinal nerve fiber layer thickness

Preoperative and postoperative measurements of the GCC and RNFL thicknesses are detailed in Table 2. Statistical analysis showed no significant differences between preoperative and postoperative values at any follow-up time point, indicating that the procedure did not exacerbate pre-existing optic nerve structural damage.

**Table 2:** Ganglion cell complex (GCC) and retinal nerve fiber layer (RNFL) thickness (μm) in patients with PSS before and after CLASS surgery.

**Patient**	**Parameter**	**Preoperative**	**Postoperative (6 months)**	**Postoperative (12 months)**	**Postoperative (18 months)**
1	GCC (μm)	68.46	66.79	66.32	65.63
	RNFL (μm)	68.45	68.25	67.28	65.88
2	GCC (μm)	72.85	71.79	68.89	68.74
	RNFL (μm)	70.45	69.35	68.89	68.77
3	GCC (μm)	75.32	75.36	74.25	72.35
	RNFL (μm)	73.29	72.48	71.25	71.25
4	GCC (μm)	63.64	63.52	63.25	63.26
	RNFL (μm)	61.48	61.20	61.24	60.85
5	GCC (μm)	65.45	66.26	65.58	64.52
	RNFL (μm)	63.34	62.24	62.85	60.52
6	GCC (μm)	66.56	66.28	65.29	65.25
	RNFL (μm)	64.54	64.26	64.25	64.50

### 3.4 Complications

Several postoperative complications were noted, all of which were mild and manageable (Table 3). One eye developed a shallow anterior chamber with over-filtration, which resolved with conservative pressure patching. Peripheral anterior synechiae (PAS) formed in two eyes and were successfully addressed with Nd:YAG laser. Transient hypotony (IOP < 10 mmHg) occurred in three eyes, with spontaneous recovery within three days. Additionally, one case exhibited mild anterior chamber exudation that subsided promptly with intensified topical corticosteroid treatment.

**Table 3:** Incidence and management of postoperative complications.

**Complication**	**Incidence (eyes)**	**Key features**	**Intervention**	**Resolution**
Shallow anterior chamber	1	Overfiltration with prominent bleb (POD 1)	Pressure patching	Resolved within 48 h
Peripheral anterior synechiae (PAS)	2	Superior angle (routine follow-up)	Nd:YAG laser	Successfully lysed, no recurrence
Transient hypotony	3	IOP 6–8 mm Hg (POD 1)	Observation	IOP normalized in ≤ 3 days
Anterior chamber reaction	1	Mild exudation with shallow chamber	Topical steroid intensification	Cleared within 24 h

### 3.5 Status of filtering structures

**Figure 2:**
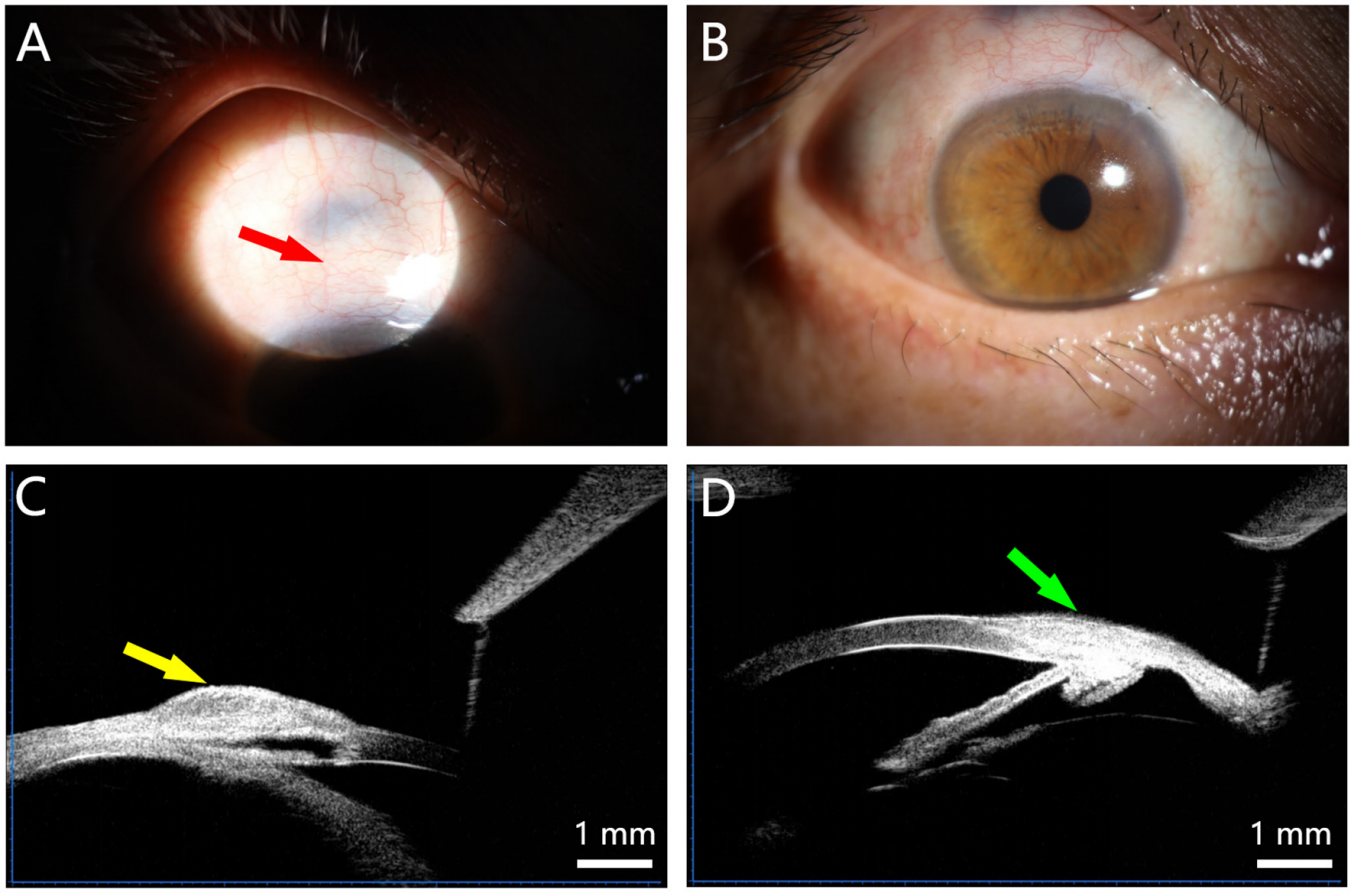
Representative postoperative imaging of the filtration site. **(A, B)** Slit-lamp photographs showing localized, elevated conjunctival filtering blebs with mild surface scarring at 12 months postoperatively. **(C)** Ultrasound biomicroscopy (UBM) image demonstrating a well-defined, intact intrascleral cavity (scleral pool, arrow). **(D)** UBM image from a different patient at 6 months postoperatively, showing loss of the discernible scleral pool architecture.

The integrity of the surgical filtration pathways was evaluated clinically and by UBM. At 12 months, all eyes maintained functional, albeit variably scarred, conjunctival filtering blebs (Figures 2A, B). UBM imaging confirmed the presence of a patent intrascleral cavity (scleral pool) in five eyes (Figure 2C). One eye showed loss of the discernible scleral pool architecture by 6 months (Figure 2D); interestingly, this eye maintained satisfactory IOP control, suggesting alternative or compensatory aqueous outflow pathways.

### 3.6 Visual field

Serial visual field testing demonstrated remarkable stability in both global (Mean Deviation, MD) and local (Pattern Standard Deviation, PSD) indices throughout the 18-month follow-up period (Table 4). Individual patient data showed minimal fluctuation, with no clinically meaningful progression observed in any case. Notably, even in Patient 1, who presented with moderate-to-severe baseline visual field loss (*MD* = −8.23 dB, *PSD* = 6.58 dB), the parameters remained stable postoperatively. Given the limited sample size, formal statistical analysis was underpowered to detect small changes; however, the consistent lack of trend toward worsening across all time points strongly suggests that CLASS surgery did not exacerbate pre-existing glaucomatous visual field damage.

**Table 4:** Visual field indices (mean deviation and pattern standard deviation) in patients with PSS before and after CLASS surgery.

**Patient**	**Parameter**	**Preoperative (0 d)**	**Postoperative (6 months)**	**Postoperative (12 months)**	**Postoperative (18 months)**
1	MD (dB)	−8.23	−8.42	−8.17	−8.21
	PSD (dB)	6.58	6.61	6.42	6.42
2	MD (dB)	−1.95	−1.92	−1.92	−1.93
	PSD (dB)	1.61	1.59	1.57	1.59
3	MD (dB)	−1.05	−1.05	−0.94	−1.02
	PSD (dB)	1.56	1.56	1.52	1.52
4	MD (dB)	−1.85	−1.84	−1.45	−1.52
	PSD (dB)	2.84	2.73	2.65	2.67
5	MD (dB)	−2.22	−2.18	−2.42	−2.38
	PSD (dB)	2.13	2.09	2.23	2.23
6	MD (dB)	−4.10	−4.05	−4.08	−4.05
	PSD (dB)	3.54	3.51	3.57	3.52

## 4 Discussion

CLASS represents a refined evolution in non-penetrating glaucoma surgery. By utilizing CO_2_ laser energy to precisely ablate the deep sclera and the outer wall of Schlemm's canal, it reduces aqueous outflow resistance while creating an intrascleral reservoir. This design facilitates dual-channel aqueous drainage: part of the aqueous humor is diverted subconjunctivally, while another portion may permeate into the suprachoroidal space through the thinned scleral bed. The procedure's non-penetrating nature, which avoids entry into the anterior chamber, minimizes intraocular manipulation and the associated inflammatory response, thereby reducing the risk of complications such as hypotony, shallow anterior chamber, and excessive fibrosis compared to traditional trabeculectomy.

In this pilot case series, we applied CLASS surgery to six patients with medically refractory PSS. The results demonstrate a significant and sustained reduction in intraocular pressure (IOP) over an 18-month follow-up period, with all eyes achieving IOP within the target range (10–21 mmHg). Notably, structural parameters—ganglion cell complex (GCC) and retinal nerve fiber layer (RNFL) thickness—remained stable, indicating no progression of optic neuropathy postoperatively. This structural preservation is particularly crucial in PSS, where the treatment goal extends beyond merely controlling episodic IOP spikes to preventing cumulative optic nerve damage during disease quiescence.

It is important to consider the unique pathophysiology of PSS when interpreting surgical outcomes. Unlike glaucoma's with chronically elevated IOP, PSS is characterized by recurrent inflammatory episodes with acute IOP elevation, interspersed with normotensive intervals. The inflammatory component is typically milder and less prone to causing posterior synechiae or rapid visual field loss. Consequently, the timing of assessment is critical; data collected exclusively during quiescent phases may not accurately reflect surgical efficacy in controlling disease activity. In this study, IOP was monitored serially to capture readings during both active and inactive phases, and long-term structural parameters (GCC, RNFL) were emphasized as more stable indicators of overall disease control. This comprehensive approach, coupled with an extended 18-month follow-up, strengthens the validity of our conclusion that CLASS surgery provides effective and durable management for refractory PSS.

Our study also informs the safety profile of CLASS in PSS. The single case of shallow anterior chamber with hypotony observed is considered potentially related to occult microtrauma to the ciliary body or anterior chamber angle, possibly caused by deeper-than-intended laser energy penetration during surgery. To mitigate this risk in future procedures, technical refinements such as incorporating intraoperative gonioscopy or ultrasound biomicroscopy for real-time monitoring could be valuable. When approaching critical anatomical structures, employing a more precise pulsed laser mode with optimized parameters is advisable. Furthermore, the use of adjustable sutures or viscoelastic agents in the early postoperative period may help maintain anterior chamber depth. Overall, the complication rate in this series remained low, supporting the relative safety of the procedure.

Given the established association of PSS with viral etiologies (cytomegalovirus, herpes simplex virus) (10), the theoretical risk of laser- or surgery-induced viral reactivation warrants consideration. Although not observed in our series or specifically reported for CLASS, this potential calls for vigilance, underscores the need for thorough preoperative evaluation, and raises the question of whether perioperative antiviral prophylaxis could benefit virus-associated cases. While our findings align with reports of CLASS's efficacy and safety in primary open-angle glaucoma (11), direct outcome comparisons are constrained by differences in study populations and endpoints. The strength of this work lies in its focused application to refractory PSS and its comprehensive, medium-term assessment of both functional (IOP, visual fields) and structural (GCC, RNFL) outcomes.

The primary limitations of this study are its small sample size and the non-controlled, single-center design, which limit the statistical power and generalizability of the findings. As a pilot series, it serves as proof of concept rather than definitive evidence. Future research should involve larger, multicenter, prospective studies with longer follow-up to validate these preliminary results. Randomized controlled trials comparing CLASS with other surgical modalities (trabeculectomy, glaucoma drainage devices) specifically in PSS patients would be invaluable to establish its comparative effectiveness and optimal role in the treatment algorithm.

## 5 Conclusion

In conclusion, this preliminary case series suggests that CLASS surgery is a promising and safe surgical option for patients with Posner-Schlossman Syndrome who have inadequate IOP control despite maximal medical therapy. It provides effective IOP reduction, preserves optic nerve structure, and is associated with a manageable complication profile. While larger studies are needed to confirm its long-term efficacy and address questions such as viral reactivation risk, CLASS presents a valuable minimally invasive alternative in the surgical management of this complex condition.

## Data Availability

The raw data supporting the conclusions of this article will be made available by the authors, without undue reservation.
